# Exploring the Relationship Between Professional Identity and Psychological Resilience of Organ Donation Coordinators in Zhejiang Province (China)

**DOI:** 10.3389/fpubh.2021.659871

**Published:** 2021-07-06

**Authors:** Wan Shu, Bing-yu Xing, Wei-xiu Ruan, Li-yan Gao, Qun-fang Miao

**Affiliations:** ^1^Medical School, Hangzhou Normal University, Hangzhou, China; ^2^Management Center of Human Organ Donation, Zhejiang, China

**Keywords:** human organ donation coordinator, professional identity, psychological flexibility, relevance, Zhejiang

## Abstract

**Background:** An organ donation coordinator plays an important role in the process of organ donation and transplant. Therefore, investigating and analyzing the current situation in organ donation and examining the correlation between professional identity and psychological resilience of human organ donation coordinator, provides a reference for promoting stable development of organ donation.

**Methods:** A total of 48 coordinators of organ donation in Zhejiang Province were recruited for the study by using the method of convenience sampling. The psychological resilience scale and professional identity questionnaire were used to collect data.

**Results:** The results revealed that the total average score of the professional identity of organ donation coordinators was 34.92 ± 8.57. Compared with the median professional identity score of 34.50, the professional identity of the coordinator in this survey was at a moderate level. The total average score of psychological resilience was 64.44 ± 11.91. There was a significant positive correlation between the professional identity of the coordinator and the total score of psychological resilience (*r* = 0.641, *P* < 0.01).

**Conclusion:** The professional identity and psychological resilience of the coordinators in Zhejiang Province were found to be in the middle level and the higher the psychological resilience score, the stronger the professional identity of the coordinators. It is important to improve the level of psychological resilience among organ donation coordinators to enhance their professional identity.

## Introduction

According to China Human Organ donation Management Center data, as of February 18, 2020, China had donated 80,800 organs, indicating that the Chinese organ donation system has made considerable progress. However, the industry is still facing an imbalance between supply and demand, with the current ratio of supply and demand being 1:30 (1). Increasing the rate of organ donation is a problem worldwide for the development of organ donation. Spain has the highest rate of organ donation in the world, and based on its experience, the shortage of organ donation is not only related to a lack of suitable donors but also the failure to successfully convert potential donors into actual donors. In Spain, the human organ donation coordinator has over the years played an important role in the reduction of the anti-donation rate from 30% (the 1990s) to 15.3% (2013) (2). In Poland, the hiring of organ donation coordinators improved the number of potential organ donors by 100%, and the number of actual organ donors was increased by 113% (2014) (3). The Human organ donation coordinator (hereinafter referred to as the coordinator) identifies potential organ donors, collects clinical information, and communicates with the families of the donors to promote the smooth development of organ donation (4). Besides, the role of the coordinator is to increase the rate of organ donation (5). When the families of donor volunteers face the great pain of bereavement, the coordinator has to urge the family members to make a decision and bid farewell within a limited time. The coordinator needs to have a strong psychological bearing capacity when facing the loss of life and the breakdown of the family.

Because of the growing demand for organ donations, China faces a shortage of organ donation coordinators. As of May 2019, there were about 2,200 coordinators in China, it is about 1–2 coordinators per million people, far less than the current rate of 7–12 coordinators per million people in the United States (6). The short time for an organ transplant and the unpredictability of life makes the work of the coordinator stressful. The National Health and Family Planning Commission in 2013 issued the regulations on the staffing of coordinators and clearly stated that coordinators must have a medical professional background, which indicates that China has carried out specific work in strengthening the professional literacy of coordinators, but related research shows that coordinators are experiencing varying degrees of anxiety, depression, poor sleep quality (7), associated with work pressure. The overall stress level is high because of low social attention, unclear career direction (8), and high mobility in staffing (9). Compared with the more mature experience in other countries, the position of coordinators should be filled by people who understand both medicine and ethics, psychology, and a good state of mind. It can be seen that there is a gap between the great values of the organ donation coordinator and the real dilemma that the coordinator faces; therefore, how to maintain the good professional competence of the coordinator under high pressure is an important topic worth exploring.

The primary component of the talent quality in professional competence is professional identity. Professional identity refers to the view of the goal, social value, and other factors of the occupation by an individual (10). Research shows that professional identity has a positive effect on the career development of coordinators, and it is an internal incentive factor (4). Higher professional identity makes coordinators more adaptable to their work and has also a direct effect on job satisfaction. The professional identity of the coordinator is the self-affirmation of the coordination work. When facing the special pressure situation in the coordination work, the internal acceptance and self-affirmation of the work can enable the coordinator to give better play to improve self-efficacy. It is a positive, active, and conscious psychological experience.

The positive psychological experience of professional identity needs the support of positive psychological quality. Psychological resilience refers to the behavior or a series of psychological reactions made by the subject in the face of changes in the external environment (11). Psychological resilience can relieve work stress, which is closely related with physical and mental health and job burnout (12), the key factor to deal with stressful situations, which means that the coordinator with higher psychological resilience can be resilient under pressure and grow up after setbacks (13). This study aimed to examine the correlation between professional identity and psychological resilience among organ donation coordinators. We hypothesized that a higher psychological resilience score correlates with a stronger professional identity among the coordinators.

## Materials and Methods

### Participants

This study was approved by the Zhejiang Human Organ Donation Management Center. A total of 48 human organ donation coordinators were recruited by the method of convenience sampling during the conference training of human organ donation coordinators in Zhejiang Province in November 2019. The inclusion criteria were as follows: possess a certificate of organ donation coordinator and have worked as a human organ donation coordinator for more than 1 year. The exclusion criteria were the person who is unwilling to accept the questionnaire.

### Procedure

We used an anonymous questionnaire, which was distributed and recovered on-site, and used unified instructions to guide the subjects to fill in the questionnaire. In this study, a total of 50 questionnaires were distributed and 48 valid questionnaires were recovered (participation rate 96%). Since the sample size is not particularly large, there may be selection bias.

### Measures

A general demographic questionnaire was distributed among the study participants. The contents of the questionnaire included were the following: gender, age, family, location, nationality, religion, education, marital status, previous occupation, mode of employment (full-time or part-time coordinator), whether they are volunteers registered for the body (organ) donation and their willingness to donate remains (organs). The professional identity scale was developed by TylerD and McCallum (14) and comprised 10 items. The total score for each of the participants was between 10 and 50 based on a Likert5 score. Gao Hong (15) applied this scale in the Chinese language showing that its internal consistency coefficient reached 0.937, and the questionnaire had good reliability. The higher the score, the higher the level of professional identity. The internal consistency coefficient of the scale is 0.937, which has good reliability and validity (15). The Chinese version of CD-RISC, translated and revised by Xiao Nan and Zhang Jianxin (16), was used and included three dimensions of tenacity, strength, and optimism. The scale is comprised of 25 items with scores in the range of 0–100 based on a Likert5 scale, the higher the score, the higher the level of psychological resilience. A score below 60 is poor, 61 ~ 69 is average, 70 ~ 79 is good, and ≥ 80 is excellent. This scale effectively evaluates the coping ability of an individual when faced with frustration, and its internal consistency coefficient α is 0.873 (16).

### Data Analysis

EpiData3.0 software was used to sort out the data, we established a database and implemented double input. Statistical analysis used the IBM SPSSS version 23.0. The statistical description of measurement data was expressed by mean and SD. Counting data were expressed in terms of frequency and percentage. Descriptive analysis, *t*-test, and Pearson's correlation analysis were used in this study.

### Ethics

The studies involving human participants were reviewed and approved by the Ethics Committee of Hangzhou Normal University. The patients/participants provided their written informed consent to participate in this study.

## Results

### Demographic Characteristics

A total of 48 human organ donation coordinators from Zhejiang Province were included in the study. Socio-demographic data were included: the mean age of the participants was 37.60 ± 7.53 years; age range was between 23 and 52 years old; 37.5% (*n* = 18) were males, 62.5% (*n* = 30) were females; about the place of birth: 8.3% came from rural areas, 22.9% came from counties and towns, 68.8% came from cities; 100% were Han nationality; 97.9% had no religious belief, 2.1% had religious belief; 2.1% had a high school degree or less, 4.2% had a technical secondary school education level, 91.6% had a bachelor's degree/junior college, and 2.1% had a master's degree; the occupations before becoming coordinators: 8.3% were doctors, 45.8% were nurses, 4.2% were technicians or pharmacists, 41.7% were 20 others; 68.8% were permanent workers, 8.3% were personnel agents, 20.8% were contract workers, 2.1% were others (including temporary workers); 45.8% were Full-time employees, 54.2% were part-time employees; whether to register volunteers for body (organ) donation: 64.6% said yes, 35.4% said no; attitude toward donating body (organ) in the future: 8.3% were unwilling, 29.2% were unclear, 62.5% were more willing.

### Factors Influencing Professional Identity of Human Organ Donation Coordinators

Statistical analysis was used to determine the relationship between professional identity scores and demographic characteristics of human organ donation coordinators. Univariate analysis showed that there were no significant differences between professional identity scores and demographic characteristics, including gender, age, family location, nationality, education level, marital status, the occupations before becoming coordinators, mode of employment (full-time or part-time job) (*P* > 0.05), as shown in Table 1.

**Table 1:** Comparison of professional identity scores of different individual characteristics of human organ coordinators (*n* = 48).

**Characteristics**	**Mean ± SD**	***t*-value/ANOVA**	***P-value***
**Gender**			
Male (*n* = 18)	37.78 ± 6.35	2.017	0.050
Female (*n* = 30) **Age**	33.20 ± 9.35		
≤ 25 (*n* = 3)	31.33 ± 12.34	0.649	0.846
26–30 (*n* = 6)	35.33 ± 7.97		
31–35 (*n* = 11)	36.91 ± 7.33		
36–40 (*n* = 14)	34.57 ± 10.39		
≥41 (*n* = 14) **Family location**	34.29 ± 7.86		
Rural areas (*n* = 4)	43.50 ± 7.33	0.417	0.979
Counties and towns (*n* = 11)	31.91 ± 7.70		
Cities (*n* = 33) **Nationality**	34.88 ± 8.49		
The Han nationality (*n* = 48)	34.92 ± 8.57		
Others (*n* = 0) **Level of education**	0		
High school degree or less (*n* = 1)	34.00 ± 0.00	0.493	0.951
Technical secondary school education level (*n* = 2)	30.00 ± 7.07		
A bachelor's degree/junior college (*n* = 44)	35.00 ± 8.77		
A master's degree or above (*n* = 1)	42 ± 0.00		
**Marital status**			
Single (*n* = 10)	32.80 ± 7.50	−0.965	0.361
Married (*n* = 38)	35.47 ± 8.84		
Widowed (*n* = 0) **The occupations before becoming coordinators**	0		
Doctors (*n* = 4)	40.75 ± 2.22	0.898	0.598
Nurses (*n* = 22)	34.73 ± 10.35		
Technicians or pharmacists (*n* = 2)	39.50 ± 7.78		
Others (*n* = 20) **Employment modality**	33.50 ± 6.92		
Permanent workers (*n* = 33)	34.61 ± 8.05	0.809	0.690
Personnel agents (*n* = 4)	31.00 ± 12.44		
Contract works (*n* = 10)	37.60 ± 9.32		
Others (including temporary workers) (*n* = 1) **Mode of employment**	34.00 ± 0.00		
Full-time (*n* = 22)	34.32 ± 9.38	−0.435	0.096
Part-time (*n* = 26) **Whether to register volunteers for body (organ) donation?**	35.42 ± 7.99		
Yes (*n* = 31)	34.42 ± 8.50	−0.531	0.815
No (*n* = 17) **Attitude toward donate body (organ) in the future**	35.82 ± 8.89		
Unwilling (*n* = 4)	31.50 ± 12.01	1.194	0.332
More reluctant (*n* = 0)	0		
Unclear (*n* = 14)	33.79 ± 8.27		
More willing (*n* = 7)	35.57 ± 7.97		
Willing (*n* = 23)	36.00 ± 8.68		

### Professional Identity and Psychological Resilience Scores

The average scores of professional identity and psychological resilience of human organ donation coordinators were 34.92 ± 8.58 and 64.44 ± 11.92, respectively. The professional identity and psychological resilience scores are shown in Table 2.

**Table 2:** The scores of professional identity and psychological resilience of coordinators (*n* = 48).

**Items**	**Scores**
Professional identity scores	34.92 ± 8.58
Psychological resilience total scores	64.4 ± 11.92
Tenacity	32.52 ± 7.31
Strength	21.15 ± 3.72
Optimism	10.77 ± 2.23

### The Correlation Between Professional Identity and Psychological Resilience of Organ Donation Coordinators

Pearson's correlation analysis showed that the professional identity of the human organ donation coordinator was positively correlated with psychological resilience, including tenacity, strength, and optimism (all *P* < 0.01), as shown in Table 3.

**Table 3:** Correlation analysis between professional identity and psychological resilience of coordinators (*n* = 48).

Variable	**Psychological resilience**	**Tenacity**	**Strength**	**Optimism**
Professional identity	0.641^**^	0.536^**^	0.594^**^	0.677^**^

** *Significant at the 0.01 level (two-tailed)*.

### Differences in Psychological Resilience Between the High and the Low Professional Identity Groups of Coordinators

Using the median professional identity score of 34.5, we compared the differences in the psychological resilience scores between the high score (≥ 34.5) and the low score (< 34.5) groups. The psychological resilience score in the high score group was significantly higher when compared with the low score group (*P* < 0.01) as shown in Table 4.

**Table 4:** Differences in psychological resilience between the high and low professional identity groups of coordinators.

**Professional identity**	**Tenacity**	**Strength**	**Optimism**	**Psychological resilience**
Low score group (*n* = 24)	29.25 ± 7.19	18.96 ± 3.62	9.54 ± 1.79	57.75 ± 10.91
High score group (*n* = 24)	35.79 ± 5.93	23.33 ± 2.28	12.00 ± 1.96	71.13 ± 8.82
*t*-value/ANOVA	−3.440	−5.014	−4.538	−4.669
*P-value*	0.001	0.001	0.001	0.001

## Discussion

### The Professional Identity of Human Organ Donation Coordinator in Zhejiang Province Is in the Middle Level

To support more patients with organ failure, China officially launched the organ donation pilot project in 2010, this led to the emergence of a career as a human organ donation coordinator. Being a new profession, there are only about 2,200 registered organ donation coordinators in China, who are unevenly distributed in various provinces and cities. Recent research shows that coordinators are faced many challenges and difficulties in their work (17). The appropriate proportion of organ matching is only 1–2%. A qualitative study on the work experience of coordinators in the United States (18) showed that work-related stress among coordinators was linked to environmental factors and individual differences. Environmental factors mainly include social support and individual participation in decision-making, while individual differences include adaptability, tenacity, and poor personality (8). Based on the local social and cultural considerations, the non-recognition of the mainstream values of the Chinese society, the formulation of laws and regulations related to organ transplant and donation, the lack of brain death criteria, and other related factors have caused work pressure among the coordinators.

In this study, the total professional identity score of the coordinators was 34.92 ± 8.57, and the median score was 34.5, which was a middle-level score and consistent with previous research findings reported in China (4). The so-called “professional identity” is to identify whether the occupation is valuable, meaningful, and can find fun in it. The coordinator often needs 24 h on standby, they face desperate people at work, so it is normal to be rejected and not understood. The loss of the family of the donor cannot be offset by the professional identity resulting from the completion of organ donation, which puts the coordinator in a very painful position. Research confirmed (19) that the coordinator will be exposed to pain and sadness for a long time during their work, which will lead to compassion fatigue. Related studies have shown that empathy fatigue negatively predicts work input (20). The coordinator who lacks social support is also asked to give certain social support to family members and patients at work, which increases the negative factors and powerlessness of the psychological stability of the coordinator. The level of the professional identity of coordinators is affected by several factors, such as the work itself, social evaluation, etc. These factors affect the stability of the coordinator team.

### Higher Psychological Resilience of Coordinators Increases Professional Identity

Psychological resilience is a positive personality trait that helps individuals cope with stress. In essence, it is a “protective factor”; therefore, individuals with high psychological resilience have a stronger ability to adapt to adversity and overcome life difficulties (21). The results showed that psychological resilience decreases work stress, promotes physical health, and improves professional identity (12, 22, 23). In addition, individuals with higher mental resilience have a relatively higher professional identity. The results also showed that the psychological resilience score of organ donation coordinators was at a medium level (64.44 ± 11.92), and there was a significant positive correlation between psychological resilience and professional identity (*P* < 0.01). This suggests that psychological resilience may be one of the factors that improve professional identity. Thus, improving the psychological resilience of coordinators may enhance their professional identity. In addition, we found that psychological resilience scores in the high professional identity score group were significantly higher than those in the low score group. The psychological resilience scale measures three dimensions: optimism, tenacity, and strength, and they were significantly correlated with the scores of professional identity (*P* < 0.01): the work of coordinator is highly sensitive as it relates to life and death; hence, high confidence is required to overcome all kinds of difficulties, they need the support of optimism in the optimistic dimension; the social attention and support received by the coordinator is insufficient, they require strong professional beliefs in tenacity dimension; as for the dimension of strength, facing the death and the inability of a family member to refuse to donate, the coordinator need to have a sense of strength. That is, the three dimensions of psychological resilience affect the acquisition of the professional identity of coordinators. The particularity of the coordinator profession requires a good psychological coping mechanism to promote organ donation. The coordinator is expected to maintain a stable mindset to enable them to adapt to adversity and overcome difficulties (21).

In this survey, there is no significant statistical difference between demographic sociological factors and professional identity, which is consistent with the study of He zhongxiang (4), which may be related to the limited sample size of this survey. This study shows that the rate of organ donation of the coordinator was 62.5%, which is higher than that reported for ordinary people in Zhejiang Province (24). This shows that the coordinator has more understanding of the greatness of the donation cause and the well-being of the people, with their behavior to identify and support the donation cause.

## Limitations

In this study, 48 organ donation coordinators in Zhejiang Province were enrolled; hence, it may contain selection bias. The application of these results to the 2,200 coordinating groups in China should be done cautiously. However, due to the small number of coordinators themselves, this study takes Zhejiang Province as an example to study, so the research results still have a certain reference value.

## Conclusion

This study reveals that the good psychological resilience of organ transplant coordinators may be a factor contributing to professional identity. Follow-up research explores the relevant factors that affect the professional identity of the coordinator from the perspective of qualitative research and promotes the professional identity of the coordinator by improving the psychological flexibility of the coordinator to better promote the development of donation.

## Data Availability Statement

The raw data supporting the conclusions of this article will be made available by the authors, without undue reservation.

## Author Contributions

WS: conceptualization, formal analysis, and writing an original draft. BX: investigation. WR and QM: resources. LG: supervision. LG and QM: review and editing. All authors contributed to the article and approved the submitted version.

## Conflict of Interest

The authors declare that the research was conducted in the absence of any commercial or financial relationships that could be construed as a potential conflict of interest.
